# The Role of Carbohydrate Response Element Binding Protein in Intestinal and Hepatic Fructose Metabolism

**DOI:** 10.3390/nu9020181

**Published:** 2017-02-22

**Authors:** Katsumi Iizuka

**Affiliations:** 1 Department of Diabetes and Endocrinology, Graduate School of Medicine, Gifu University, Gifu 501-1194, Japan; kiizuka@gifu-u.ac.jp; Tel.: +81-58-230-6564; Fax: +81-58-230-6376; 2 Gifu University Hospital Center for Nutritional Support and Infection Control, Gifu 501-1194, Japan

**Keywords:** carbohydrate response element binding protein, ChREBP, glycolysis, fructolysis, Glut5/SLC2A5, ketohexokinase, fructose

## Abstract

Many articles have discussed the relationship between fructose consumption and the incidence of obesity and related diseases. Fructose is absorbed in the intestine and metabolized in the liver to glucose, lactate, glycogen, and, to a lesser extent, lipids. Unabsorbed fructose causes bacterial fermentation, resulting in irritable bowl syndrome. Therefore, understanding the mechanisms underlying intestinal and hepatic fructose metabolism is important for the treatment of metabolic syndrome and fructose malabsorption. Carbohydrate response element binding protein (ChREBP) is a glucose-activated transcription factor that controls approximately 50% of de novo lipogenesis in the liver. ChREBP target genes are involved in glycolysis (Glut2, liver pyruvate kinase), fructolysis (Glut5, ketohexokinase), and lipogenesis (acetyl CoA carboxylase, fatty acid synthase). ChREBP gene deletion protects against high sucrose diet-induced and leptin-deficient obesity, because *Chrebp*^−/−^ mice cannot consume fructose or sucrose. Moreover, ChREBP contributes to some of the physiological effects of fructose on sweet taste preference and glucose production through regulation of ChREBP target genes, such as fibroblast growth factor-21 and glucose-6-phosphatase catalytic subunits. Thus, ChREBP might play roles in fructose metabolism. Restriction of excess fructose intake will be beneficial for preventing not only metabolic syndrome but also irritable bowl syndrome.

## 1. Introduction

Obesity and its related diseases (diabetes mellitus, fatty liver, and dyslipidemia) are now significant social and economic problems in Western countries. A number of articles have discussed the relationship between fructose consumption (especially sugar-sweetened beverages) and the incidence of obesity and related diseases [1,2,3,4,5]. Increased fructose consumption contributes to the development of obesity accompanied by glucose intolerance, fatty liver, dyslipidemia, and hyperuricemia [3]. Additionally, in experimental animals, excess fructose intake causes body weight gain and fatty liver changes [3,4,5]. However, some studies have reported that there is no correlation between fructose consumption and obesity-related diseases [1,2]. Does fructose consumption really cause metabolic syndrome?

Plasma fructose levels (~200 μM in animals and 10–70 μM in humans) are much lower than plasma glucose levels (~6 mM) [6,7]. However, plasma fructose levels are positively correlated with glycemic control [7]. Fructose has more potent cytotoxicity because of increased advanced glycation end product (AGE) production [8,9]. Thus, fructose is not as readily absorbed and is immediately converted into other metabolites, such as glucose, triacylglycerol, and lactate in the intestine and liver [10,11,12]. If excess fructose is consumed, undigested fructose can cause bacterial fermentation, resulting in abdominal pain, flatulence, and diarrhea [13]. Therefore, clarification of the regulatory mechanisms underlying intestinal and hepatic fructose metabolism will be beneficial for understanding the pathogenesis of not only obesity-related diseases, but also fructose malabsorption.

We have analyzed the role of carbohydrate response element binding protein (ChREBP) in the pathogenesis of metabolic diseases [14]. ChREBP is a glucose-activated transcription factor that regulates glucose and lipid metabolism [5,14,15,16,17]. ChREBP is abundantly expressed in the liver and intestine [18,19,20] and plays important roles in the regulation of fructose metabolism [20,21,22]. Moreover, ChREBP regulates the gene expression of proteins involved in monocarbohydrate transport, glycolysis, fructolysis, and de novo lipogenesis [20,23,24,25]. Therefore, ChREBP plays an important role in glycolysis and fructolysis. In this review, I describe glucose and fructose metabolism with special references to the roles of ChREBP. Fructose is slowly absorbed from the intestine and immediately metabolized in the liver. Considering the different roles between the liver and intestine, clarification of the mechanisms underlying both intestinal and hepatic fructose metabolism is important.

## 2. Metabolic Fate of Fructose

### 2.1. The Role of the Intestine in Fructose Metabolism

Fructose is a simple ketonic monosaccharide that is rich in fruits and honey. Fructose is used commercially in beverages for its high relative sweetness. Fructose is passively absorbed from the lower part of the duodenum and jejunum by glucose transporter 5 (GLUT5/SLC2A5) and transported into the blood by glucose transporter 2 (GLUT2/SLC2A2) [26,27]. Some studies have reported that the Michaelis constant (K_M_) of SLC2A5 for fructose is ~6 mM and that of SLC2A2 is ~11 mM [12]. In the intestine, absorption rates for fructose are much slower than those for glucose [12]. In one study in humans, ingestion of 5 or 10 grams of fructose led to 10% of the study group being diagnosed as fructose malabsorbers [28]. This number increased to 40% when 20 grams of fructose was ingested [28]. Almost 40% of patients exhibited fructose malabsorption at an intake of 25 grams, and 66% of patients at an intake of 50 grams [29]. The absorption capacity of fructose in monosaccharide form in adult rats was equivalent to 1.4–1.6 g fructose/kg body weight [30]. Acute fructose malabsorption occurred with doses greater than 2.1–2.4 g/kg body weight [30]. Moreover, fructose malabsorption is caused by defects in fructose transporters, such as SLC2A5 and SLC2A2 [12]. Intestinal fructose malabsorption causes abdominal complaints, such as abdominal pain, bloating, flatulence, and diarrhea. These symptoms are due to bacterial fermentation of unabsorbed fructose in the colon. Deletion of the gene encoding *Slc2a5* in mice fed a high fructose diet resulted in decreased fructose absorption by 75% in the jejunum and decreased serum fructose levels by 90%. Similar to fructose malabsorption in humans, the caecum and colon in high fructose diet-fed *Slc2a5*^−/−^ mice were dilated because of bacterial fermentation [31]. Thus, overconsumption of fructose causes irritable bowel syndrome in humans and animals. 

### 2.2. Role of the Liver in Fructose Metabolism

**Figure 1:**
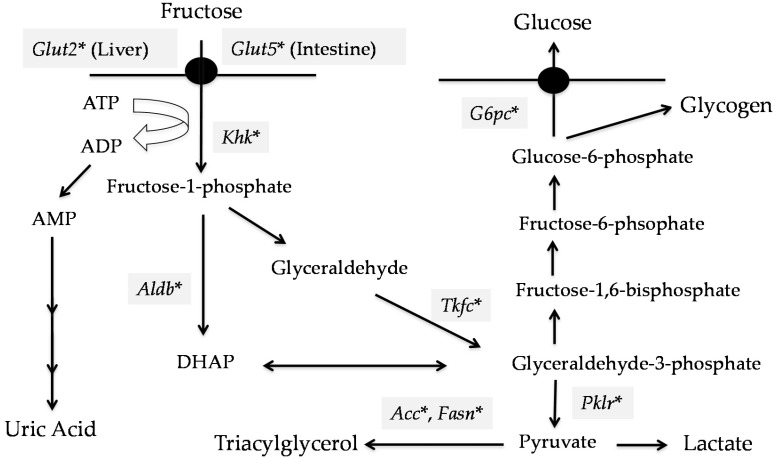
ChREBP regulates fructolytic gene expression. Fructose is transported by GLUT5 and metabolized by ketohexokinase, aldolase B, and triokinase. Dihydroxyacetone phosphate and glyceraldehyde-3-phosphate enter into the glycolytic or gluconeogenic pathway. * Genes are regulated by ChREBP [14,20,23,24,25]. *Khk*, ketohexokinase; *G6pc*, glucose-6-phosphatase catalytic subunit; *Aldb*, aldolase B; *Pklr*, pyruvate kinase, liver and reticulocyte type; *Acc*, acetyl coA carboxylase; *Fasn*, fatty acid synthase; *Tkfc*, triokinase; ChREBP, carbohydrate response element binding protein; GLUT2, glucose transporter 2; GLUT5, glucose transporter 5; DHAP, Dihydroxyacetone phosphate; ATP, adenosine triphosphate; ADP, adenosine diphosphate; AMP, adenosine monophosphate.

Conversion from fructose into glucose is limited in intestine. At lower luminal fructose concentrations in the intestine (~1 mM), ~60% of fructose is converted to glucose [12]. At higher luminal concentrations, fructose is metabolized in the liver. Portal vein fructose concentrations are 1 mM, while peripheral fructose concentrations are ~0.1 mM [12]. As *SLC2A5* expression in the liver is much lower than in the intestine, fructose in the liver is transported by SLC2A2 and phosphorylated into fructose-1-phosphate by ketohexokinase (KHK)/Fructokinase [12]. Fructolysis is much faster than glycolysis. Enzymes specific for fructose metabolism include KHK, aldolase B, and triokinase (ATP: D-glyceraldehyde 3-phosphotransferase) (Figure 1). These enzymes are highly expressed in the liver, kidney, and intestine [32]. There are two KHK isoforms, KHK-C and -A. Both can metabolize fructose, but KHK-C is considered the primary enzyme involved in fructose metabolism because of its lower K_M_ [33,34,35]. In hepatocellular carcinomas, fructolysis is much slower than in healthy hepatocytes because of a switch from KHK-C to KHK-A [35]. Thus, KHK-C, rather than KHK-A, primarily regulates fructolysis. Fructolysis bypasses the steps using glucokinase and phosphofructokinase, which are rate-limiting enzymes in glycolysis. Fructose-1-phosphate is then converted into dihydroxyacetone phosphate and glyceraldehyde via aldolase B. Glyceraldehyde is converted into glyceraldhyde-3-phosphate by triokinase. Dihydroxyacetone phosphate and glyceraldehyde-3-phosphate are identical to those in glycolysis and can enter the gluconeogenic pathway for glucose or glycogen synthesis or be further catabolized through the lower glycolytic pathway to lactate or de novo lipogenesis [11,12].

**Figure 2:**
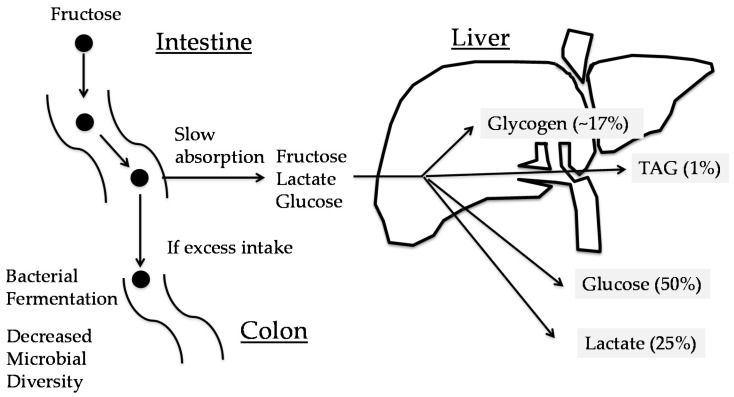
Metabolic fate of fructose. Fructose is slowly absorbed in the intestine. If excess fructose is consumed, unabsorbed fructose causes bacterial fermentation and, thereby, irritable bowel syndrome. Absorbed fructose is converted into glucose (50%), glycogen (~17%), lactate (25%), and triacylglycerol (TAG) (1%) [11].

In healthy subjects, after ingestion of a fructose load, plasma glucose and insulin levels change significantly less than those following a glucose load. Plasma fructose levels are increased to 50–500 μM [11]. Fructose is converted into glucose (28.9%–54%), lactate (~28%), glycogen (17%), and triacylglycerol (<1%) rapidly (<6 h) (Figure 2) [11]. These data suggest that the SLC2A5/SLC2A2-KHK system in the intestine and liver successfully protects against fructose toxicity. The contribution of excess fructose consumption to hyperlipidemia might be much lower in humans.

## 3. ChREBP Regulates Glycolysis and Fructolysis through Altered Gene Expression

### 3.1. Glucose Metabolism

ChREBP is a transcription factor that belongs to a family of basic helix-loop-helix leucine zipper-type transcription factors [18,19]. ChREBP and Max-like protein X (MLX) form a heterodimer that binds carbohydrate response elements (ChoREs) in the promoters of ChREBP target genes [19,36,37]. ChREBP is expressed in the liver, kidney, intestine, muscle, white adipose tissue, brown adipose tissue, and pancreatic islets [18,19,20]. In contrast, Mlx is ubiquitously expressed across tissues [19].

ChREBP has two isoforms, ChREBP-α and ChREBP-β [38]. Both ChREBP isoforms and Mlx form complexes (ChREBP-α-MLX and ChREBP-β-MLX) that regulate ChREBP target gene expression [38]. ChREBP-α is less potent than ChREBP-β. However, ChREBP-α has a low glucose inhibitory domain [38]. Under low glucose conditions, the low glucose inhibitory domain suppresses ChREBP-α transactivity [39]. In contrast, ChREBP-β is constitutively active under any glucose conditions. ChREBP-β is induced by ChREBP-α [38], and ChREBP-β suppresses ChREBP-α expression [40,41,42]. Therefore, we hypothesized that ChREBP-α and ChREBP-β serve as a sensor and amplifier for glucose signaling, respectively [14]. The ChREBP-MLX complex regulates genes related to glycolysis, lipogenesis, gluconeogenesis, transcription factors, and hormone signaling [14,20,23,24,25]. Therefore, ChREBP contributes to glucose and lipid homeostasis by regulating metabolic gene expression.

**Figure 3:**
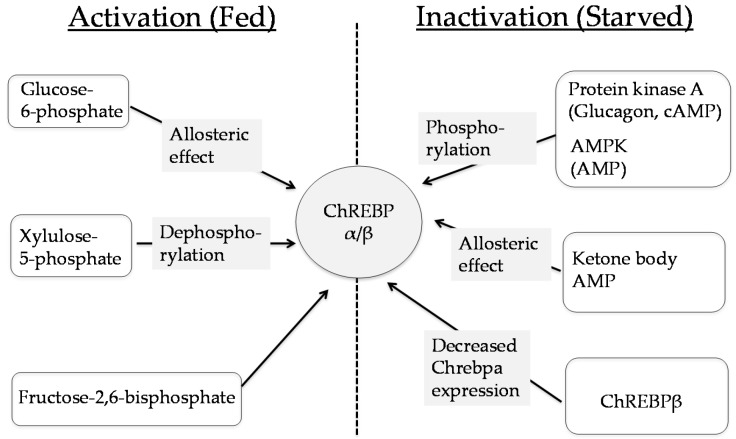
ChREBP transactivities are regulated by several factors. ChREBP is activated by glucose derived metabolites and suppressed by AMP, ketone bodies and cyclic cAMP [43,44,45,46,47,48,49,50,51,52]. AMP, adenosine monophosphate; AMPK, AMP-activated protein kinase; cAMP, cyclic AMP.

ChREBP is activated by several metabolites, such as glucose-6-phosphate, xylulose-5-phosphate, fructose-2,6-bisphosphate, and Uridine diphosphate *N*-acetylglucosamine (UDP-GlcNAc), and suppressed by adenosine monophosphate (AMP), ketone bodies and cyclic cAMP [43,44,45,46,47,48,49,50,51,52] (Figure 3). 

**Figure 4:**
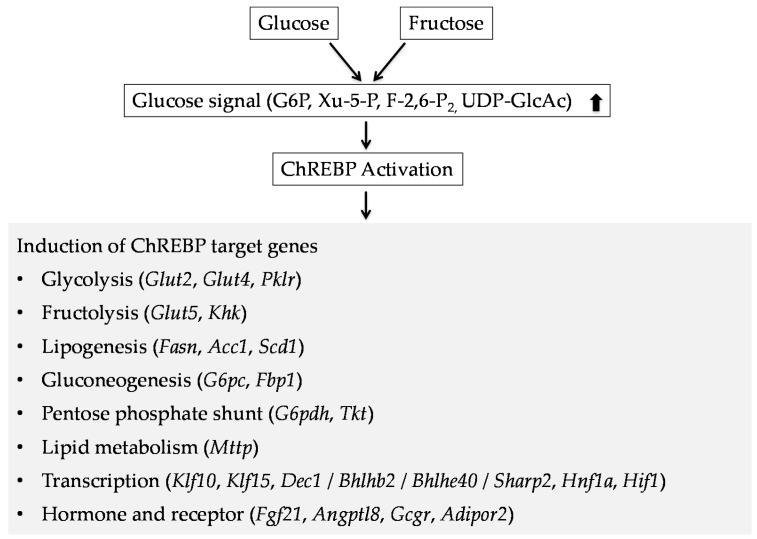
ChREBP has an important role in regulating glucose and lipid metabolism. Glucose and fructose regulate many genes expression through ChREBP activation [14,20,23,24,25]. *Glut2*, glucose transporter 2; *Glut4*, glucose transporter 4; *Pklr*, pyruvate kinase, liver and red blood cell; *Glut5*, glucose transporter 5; *Khk*, ketohexokinase; *Fasn*, fatty acid synthase; *Acc1*, acetyl coA carboxylase 1; *Scd1*, stearoyl CoA desaturase; *G6pc*, glucose-6-phosphatase catalytic subunit; *Fbp1*, fructose-1,6-bisphosphatase 1; *G6pdh*, hexose-6-phosphate dehydrogenase; *Tkt*, transketolase; *Mttp*, microsomal triglyceride transfer protein; *Klf10*, kruppel-like factor 10; *Klf15*, kruppel-like factor 15; *BHLHE40*, basic helix-loop-helix family, member E40; *Bhlhb2*, Basic helix-loop-helix domain-containing protein, class B; *Hnf1a*, hepatocyte nuclear factor 1a; *Hif1*, hypoxia inducible factor 1; *Fgf21*, fibroblast growth factor 21; *Angptl8*, angiopoietin like 8; *Gcgr*, glucagon receptor; *Adipor2*, adiponectin receptor 2.

Metabolites that can activate ChREBP are involved in the glycolytic and pentose phosphate pathways [43,44,45,46,47,48]. Glycolysis and the pentose phosphate shunt are linked to de novo lipogenesis through nicotinamide adenine dinucleotide supply and demand [23]. Glycolysis (via the tricarboxylic acid cycle) and the pentose phosphate shunt supply the substrates citrate and the reduced form of nicotinamide adenine dinucleotide for de novo lipogenesis. ChREBP regulates genes involved in the glycolytic (genes encoding liver type pyruvate kinase and Glut2), pentose phosphate (gene encoding transketolase), and de novo lipogenic (genes encoding fatty acid synthase and acetyl CoA carboxylase) pathways (Figure 4). Thus, ChREBP plays an important role in regulating hepatic glycolytic and lipogenic gene expression.

### 3.2. Fructose Metabolism

As with genes related to fructose metabolism, glucose enhances fructose absorption in the intestine. Glucose and triiodothyronine coordinately induce *SLC2A5* mRNA expression in human colon CACO2 cells [53]. Similarly, fructose and triiodothyronine coordinately induce *Slc2a5* mRNA expression in the small intestine of rats during the weaning period [54]. However, in weaning pups made hypothyroid from birth, dietary fructose can still enhance intestinal fructose uptake and *Slc2a5* mRNA expression, even though thyroxine levels in the serum are very low. Therefore, glucose and fructose primarily activate *Slc2a5* mRNA expression in vivo and in vitro.

There are a few mechanisms underlying glucose- and fructose-induced *Slc2a5* mRNA expression. ChREBP is known to regulate *Slc2a5* gene expression. *Chrebp*^−/−^ mice displayed lower *Slc2a5* mRNA levels in the intestine and liver than those in wild-type (WT) mice [20] (Iizuka K and Kato T, unpublished data). Similarly, glucose upregulates *Slc2a5* mRNA expression, and overexpression of dominant negative MLX suppresses glucose induction of *Slc2a5* mRNA in primary rat hepatocytes [23]. Similar to glucose, fructose might activate ChREBP transactivity by *O*-glycosylation (via the hexosamine pathway), phosphorylation (via xylulose-5-phosphate), and conformational change (via glucose-6-phosphate) [5,21,22,43,44,45,46,47,48]. Furthermore, fructose can increase *Slc2a5* mRNA stability through the cAMP pathway and polyadenylated-binding protein-interacting protein 2 binding [55].

Recently, some groups reported that there was an indirect pathway mediated by thioredoxin-interacting protein (TXNIP) [56,57]. TXNIP plays an important role in regulating intracellular redox state [58]. Glucose and fructose induce *TXNIP* gene expression partly through ChREBP and MondoA, an orthologue of ChREBP [59,60]. Fructose also promotes fructose uptake through the interaction between TXNIP and GLUT5/GLUT2. Consistent with this, *TXNIP* gene deletion prevented body weight gain and fatty liver caused by high fructose diet consumption.

KHK is also a gatekeeper gene that protects from increasing plasma fructose levels [5]. *Khka/c*^−/−^ mice display fructosuria and decreased adiposity and hepatic fat content [31]. One group demonstrated that there are two ChoRE regions in human *KHK* promoters (proximal, −722 to −739 bp; distal, −2902 to −2885 bp) [61]. The ChoRE in the *Slc2a5* promoter is not yet identified. However, considering that *Slc2a5* and *Khk* gene deletion both suppress fructose-induced *Chrebp* gene expression [62], metabolites derived from fructose may regulate fructolytic gene expression through CHREBP activation (Figure 1).

## 4. Chrebp Deletion Suppresses Obesity and Fatty Liver Induced by Excess Carbohydrate Feeding

Does ChREBP regulate glucose and lipid homeostasis in vivo? The answer lies in the results from *Chrebp*^−/−^ mice. *Chrebp*^−/−^ mice display several characteristic phenotypes [20]. Compared with wild type (WT) mice, *Chrebp*^−/−^ mice exhibit hepatomegaly because of hepatic glycogen accumulation and reduced white adipose tissue weights [20]. Additionally, plasma free fatty acid, ketone body, and cholesterol levels in *Chrebp*^−/−^ mice were much lower than those in WT mice [20,63].

Ob/ob mice are characterized by a leptin gene mutation and display excess dietary intake. *Chrebp* gene deletion prevents body weight gain and fatty liver by decreasing food intake [64]. The results of adenoviral short hairpin ribonucleic acid (shRNA) against *Chrebp* in ob/ob mice were similar to our results [65]. Similarly, in *Chrebp*^−/−^ mice fed a high fat/high cholesterol/high sucrose diet, body weight gain was suppressed because of decreased food intake [63]. These mice also displayed cholesterol gallstones (Iizuka K, unpublished data). In contrast, in *Chrebp*^−/−^ mice fed a high starch diet, body weight gain was similar to that in WT mice [20]. In *Chrebp*^−/−^ mice fed a high fat/low sucrose diet, similar results were also observed (Iizuka K and Takao K, unpublished data). These data indicate that *Chrebp*^−/−^ mice could not consume sucrose or fructose. Moreover, high sucrose-fed *Chrebp*^−/−^ mice displayed massive dilatation of the caecum and colon, similar to *Slc2a5*^−/−^ mice fed a high sucrose diet (Iizuka K, et al. unpublished data) [30]. As *Slc2a5* mRNA is regulated by ChREBP, the inability of *Chrebp*^−/−^ mice to consume sucrose might be partly due to decreased intestinal *Slc2a5* expression. We are now working to identify the mechanism underlying why *Chrebp*^−/−^ mice could not consume a fructose-rich diet and, particularly, the role of the intestine.

## 5. Newly-Identified Roles of ChREBP: Regulation of Sweet Preference and Hepatic Glucose Production

### 5.1. Fibroblast Growth Factor (FGF)-21

**Figure 5:**
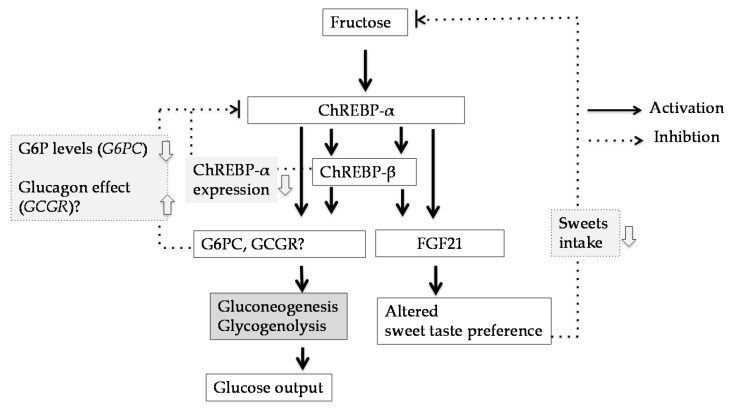
Fructose induces G6pc and Fgf21 gene expression thorugh ChREBP activation. ChREBP-α regulates ChREBP target genes expression. In turn, products of ChREBP target genes (ChREBP-β, G6pc, Gcgr, and Fgf-21) might suppress ChREBP transactivity [20,21,22,67,73]. FGF-21 suppress ChREBP transactivity by decreasing sweets intake [70,71]. GCGR might suppress ChREBP activity by enhancing glucagon effects and thereby protein kinase A activity. G6PC might suppress ChREBP activity by decreasing intracellular G6P levels. G6PC, glucose-6-phosphatase catalytic subunit; GCGR, glucagon receptor; FGF21, fibroblast growth factor-21; G6P, glucose -6-phosphate; ChREBP, carbohydrate response element binding protein.

FGF-21 is a promising therapeutic target for obesity and dyslipidemia [66]. FGF-21 is a secretory hormone induced by starvation through peroxisome proliferator-activated receptor alpha. In contrast, plasma FGF-21 levels in obese individuals are much higher those in lean individuals. We observed that ChREBP directly activated *Fgf-21* gene expression in rat hepatocytes [67]. Moreover, some studies have reported that oral glucose and fructose injections increase plasma FGF21 levels [68] and acute increase in circulating FGF-21 following fructose gavage was absent in ChREBP knockout mice [22]. In contrast, induction of ChREBP-β and its gene targets were attenuated in *Fgf-21*^−/−^ mice fed high-fructose diets [22]. After eight weeks of high-fructose diet, livers from *Fgf-21*^−/−^ mice demonstrate atrophy and fibrosis [22]. Considering that FGF-21 did not directly affect ChREBP transactivity in rat hepatocytes [68,69], probably the effect of FGf21 gene deletion on fructose induced hepatic fibrosis might be due to indirect pathway. Recently, some groups have reported that FGF-21 modulates simple sugar intake and sweet taste preference by producing an endocrine satiety signal that acts centrally to suppress sweet intake [70,71]. The liver-to-brain FGF21 axis may represent a negative feedback loop, as hepatic FGF21 production is elevated by glucose- and fructose-mediated ChREBP activation (Figure 5). 

### 5.2. Glucose-6-Phosphatase Catalytic Subunit (G6pc)

The relationship between fructose consumption and hepatic insulin resistance has been documented [3]. *G6pc* mRNA expression is decreased in *Chrebp*^−/−^ mice [20,21,64], and some studies have reported that ChREBP directly regulates *G6pc* gene expression in hepatocytes [72]. A recent report revealed that ChREBP regulated fructose-induced hepatic glucose production through increased *G6pc* expression [20]. Interestingly, in *Chrebp*^−/−^ mice, glucagon failed to stimulate glycogenolysis and, thereby, glucose production [20]. Considering that ChREBP regulates glucagon receptor (*Gcgr*) gene [23,73], not only *G6pc* but also *Gcgr* has some role in ChREBP-mediated glucose production from frcutose. Moreover, they also reported that ChREBP-β is correlated with *G6pc* expression as well as expression of the genes encoding liver pyruvate kinase and fatty acid synthase in liver biopsy samples from overnight-fasted human subjects with non-alcoholic fatty liver disease [21,74]. 

However, there is now epidemiological controversy regarding fructose consumption and insulin resistance [1,2]. A fructose intake exceeding 150 g/day in adults reduces fasting insulin sensitivity, and fructose intake exceeding 250 g/day suppresses hepatic glucose output by insulin in humans [1]. When solutions containing 25–50 g of fructose (equivalent to >500 mL high fructose corn syrup-sweetened soft drink) are consumed, >50% of healthy subjects demonstrate fructose malabsorption and, consequently, experience symptoms of abdominal pain, flatulence, and loose bowels [26,27]. Considering the difficulty of intestinal fructose absorption, whether fructose-mediated ChREBP activation in the liver contributes to hepatic glucose output regulation should be further investigated (Figure 5).

## 6. The Role of ChREBP in Fructose Metabolism

As described above, slow fructose absorption from the intestine and faster conversion from fructose into glucose is important for fructose metabolism. If fructose absorption rates were as fast as glucose, and fructolysis was regulated by a negative feedback system, plasma fructose levels would be as high as plasma glucose levels. As fructose is more potent and much faster in terms of hemoglobin A1c (HbA1c) formation [8,9], HbA1c levels and diabetic vascular complications would worsen. Therefore, although fructose can theoretically induce metabolic syndrome in high fructose-fed animal models, intestinal fructose absorption might normally be a rate-limiting barrier that protects from increasing plasma fructose concentration. ChREBP potentially regulates both intestinal and hepatic fructose metabolism. ChREBP suppression is beneficial for metabolic syndrome and obesity, and several anti-dyslipidemic and anti-diabetic drugs are known to suppress ChREBP transactivity [14]. If these drugs (for example, metformin) impact the intestine, excess sucrose and fructose intake might cause diarrhea and abdominal pain because of decreased fructose absorption. Thus, irritable bowel syndrome may be a warning symptom to prevent against excess fructose intake. A diet low in fermentable oligosaccharides, disaccharides, monosaccharides, and polyols is known to be beneficial for the treatment of irritable bowl syndrome [75]. Therefore, restriction of excess fructose intake, such as fructose-containing beverages, and lowering consumption of fermentable oligosaccharides, disaccharides, monosaccharides, and polyols will be beneficial for protecting against metabolic syndrome and irritable bowel syndrome.

## 7. Conclusions

ChREBP plays an important role in regulating fructose absorption and conversion from fructose into glucose, lactate, glycogen, and lipids. The role of ChREBP in fructose-mediated fatty liver might be very low because fructose is difficult to be absorbed in the intestine. However, chronic fructose intake might increase the efficiency of intestinal fructose absorption through intestinal Glut5 expression induced by ChREBP activation. Considering that fructose is harmful in the development of metabolic syndrome and irritable bowl syndrome, restriction of fructose intake might be important for protection against these conditions.
